# Supplementary material for: Dynein-mediated transport and membrane trafficking control PAR3 polarised distribution
Source: eLife. 2019 Jan 23;8:e40212. doi: 10.7554/eLife.40212 (PMC6358217; doi:10.7554/eLife.40212)
Supplement: Source code 1. [file elife-40212-code1.pdf]

```
macro "OocyteAnalysis Action Tool -
C000Dd0C000De0C000Dc1Dd1C000Dc0De1DffC000DfeC111D60DeeDefDfaDfbDfcDfdC111D70Db1DdfDebDecDedC11
1D0fD50DceDcfDdcDddDdeC111D0eD1fDa2DafDbdDbeDbfDccDcdDf1C111D2fD6fD79D7fD80D89D8fD9eD9fDadDaeD
e2C111D0aD0bD4fD5fD61D6aD71D7aD8eDb2DdbC111D09D0dD3fD5aD5bD78D88DbcDf0C111D0cD19D4bD69D93Dc2Df
3C111D6bD8aDd2DeaDf9C111D28De3C111D7eDd3Df6C111D18D1eD90D9dDa3Db0De6C111De5C111D4cD7bDcbDd5C11
1Dd4C111D1aD3cD84Da1C222D08Dc3Dc5De4Df4C222D56D65Da0De9Df2C222D37D47D5cD6eDf5C222D74D81D92C222
D00D40D83DacC222D75Dd7C222D29D38D4aD59Da4DdaC222De7C222DbaDd6C222D54D68D94Db3Db6C222D3bD8dD91D
c7Dd8C222D5eD99DbbDe8C222D27D46D6cD8bD9aDb5Dc6C222D77D87Db9Dc4DcaC222D01D55C222D45D66Db7Df8C33
3D03D44D51D53Db8C333D17D2eD72D82C333D64C333D02D1bD4eC333D10D12C333D16D9cC333D26Da6C333D11D36D9
8Da7C333D1dD62D7dD85D97C333D2cD3eDabDb4C333D15D57D73Da5Dc8Df7C333D07Dd9C333D23D41D43D9bC333D1c
D7cC333D13D21D22C333D14C444D04D32D3dD8cC444D4dC444D05D2aD31D33D52D6dC444D24D63D76C444D42D48D86
DaaC444D5dC444D2dD34D39D67C444D49D58Dc9C444D20D3aC444Da8C444D2bD96C444D30C444D35C555D95C555Da9
C555D25C555C666D06"{

	waitForUser("Choisir le dossier d enregistrement des tableaux de valeurs");
	dir = getDirectory("Choose a Directory");
	waitForUser("Ouvrir l image a traiter");
	Image=getImageID();
	title=getTitle();
	Stack.getDimensions(w,h,c,s,f);
	getVoxelSize(ww, hh, zz, unit);
	selectImage(title);
	run("Enhance Contrast", "saturated=0.35");
	run("Line Width...", "line=1");

	if (c == 1){
		Dialog.create("Tout d abord...");
		Dialog.addCheckbox("Analyse des membranes",true);
		Dialog.addCheckbox("Avec profil d intensite", true);
		Dialog.addCheckbox("Avec cytoplasme",true);
		Dialog.addCheckbox("Garder toutes les images ouvertes",true);
		Dialog.addNumber("Numero de l ovocyte : ", 0);
		Dialog.addNumber("Surface en pixel de la plus petite particule : ", 2);
		Dialog.addNumber("Coefficient axe Antero-posterieur : ", 0.4);
		Dialog.show();
		cortex = Dialog.getCheckbox();
		profil = Dialog.getCheckbox();
		cytoplasme = Dialog.getCheckbox();
		z = Dialog.getCheckbox();
		num_ovo = Dialog.getNumber();
		Surf_part = Dialog.getNumber();
		coef = Dialog.getNumber();
		run("Duplicate...","title=Image duplicate channels=1");
	}
	else{
		Dialog.create("Tout d abord...");
		Dialog.addCheckbox("Analyse des cortex",true);
		Dialog.addCheckbox("Avec profil d intensite", true);
		Dialog.addCheckbox("Avec cytoplasme",true);
		Dialog.addCheckbox("Garder toutes les images ouvertes",true);
		Dialog.addNumber("Numero de l ovocyte : ", 0);
		Dialog.addNumber("Surface en pixel de la plus petite particule : ", 2);
		Array1 = newArray("Canal 1","Canal 2","Canal 3","Canal 4");
		Array2 = newArray(true, false, false, false);
		Dialog.addCheckboxGroup(1,4,Array1,Array2);
		Dialog.addNumber("Coefficient axe Antero-posterieur : ", 0.4);
		Dialog.show();
		cortex = Dialog.getCheckbox();
		profil = Dialog.getCheckbox();
		cytoplasme = Dialog.getCheckbox();
		z = Dialog.getCheckbox();
		num_ovo = Dialog.getNumber();
		Surf_part = Dialog.getNumber();
		Canal1 = Dialog.getCheckbox();
		Canal2 = Dialog.getCheckbox();
		Canal3 = Dialog.getCheckbox();
		Canal4 = Dialog.getCheckbox();
		coef = Dialog.getNumber();

		if (Canal1 == true){
			Stack.setChannel(1);
			run("Duplicate...","title=Image");}
		else if (Canal2 == true){
			Stack.setChannel(2);
			run("Duplicate...","title=Image");}
		else if (Canal3 == true){
			Stack.setChannel(3);
			run("Duplicate...","title=Image");}
		else if (Canal4 == true){
```

```
                Stack.setChannel(4);
                run("Duplicate...","title=Image");}
        }

//************************************************SEPARATION DES PARTIES DE
L'OVOCYTE*****************************************************************************************
***********

        selectWindow("Image");
        Image = getImageID();
        run("Duplicate...","title=Image-1 duplicate channels=1");
        run("Threshold...");
        setAutoThreshold("Default dark");
        waitForUser("Choix du seuillage de l image!");
        setOption("BlackBackground", true);
        run("Convert to Mask");
        run("8-bit");


        imageCalculator("AND create", "Image","Image-1");
        rename("Image3");

        setTool("point");
        waitForUser("Placer un 1er point: \n 1/ Extremite marge anterieur A");
        run("Measure");
        run("Add to Manager");
        Xa = getResult("X");
        Ya = getResult("Y");

        waitForUser("Placer un 2eme point: \n 2/ Extremite marge anterieur B");
        run("Measure");
        run("Add to Manager");
        Xb = getResult("X");
        Yb = getResult("Y");

        waitForUser("Placer un 3eme point: \n 3/ Posterieur ");
        run("Measure");
        run("Add to Manager");
        roiManager("Show All");
        Xp = getResult("X");
        Yp = getResult("Y");

        Xm = Xa+(Xb-Xa)/2;
        Ym = Yb+(Ya-Yb)/2;

        makeLine(Xm/ww, Ym/ww, Xp/ww, Yp/ww);
        run("Add to Manager");
        roiManager("Show All");

        apm = (Yp-Ym)/(Xp-Xm);
        bpm = (Yp+Ym-apm*(Xp+Xm))/2;

        Yn = Yp + (Ym-Yp)*coef;
        Xn = Xp + (Xm-Xp)*coef;

        abn = (Yn-Yb)/(Xn-Xb);
        bbn = (Yn+Yb-abn*(Xn+Xb))/2;

        aan = (Yn-Ya)/(Xn-Xa);
        ban = (Yn+Ya-aan*(Xn+Xa))/2;

        makeLine((-bbn/abn)/ww, 0, ((w-bbn)/abn)/ww, w/ww);
        run("Add to Manager");
        roiManager("Show All");
        makeLine((-ban/aan)/ww, 0, ((w-ban)/aan)/ww, w/ww);
        run("Add to Manager");
        roiManager("Show All");


        Ym1 = Yp + (Ym-Yp)*2/3;
        Xm1 = Xp + (Xm-Xp)*2/3;
        Ym2 = Yp + (Ym-Yp)/3;
        Xm2 = Xp + (Xm-Xp)/3;

        Xa1 = Xa + (Xm1-Xm);
        Ya1 = Ya + (Ym1-Ym);
        Xa2 = Xa1 + (Xm2-Xm1);
        Ya2 = Ya1 + (Ym2-Ym1);
```

```
        Xb1 = Xb +(Xm1-Xm);
        Yb1 = Yb + (Ym1-Ym);
        Xb2 = Xb1 + (Xm2-Xm1);
        Yb2 = Yb1 + (Ym2-Ym1);

        makeLine(Xa1/ww,Ya1/ww,Xb1/ww,Yb1/ww);
        run("Add to Manager");
        makeLine(Xa2/ww,Ya2/ww,Xb2/ww,Yb2/ww);
        run("Add to Manager");

        // calcul de l'aire et de la somme des niveaux de gris du cytoplasme
        run("Clear Results");
        Area_Cyto=0;
        RawIntDen_Cyto=0;
        IntDen_Cyto=0;
        setTool("polygon");
        waitForUser("Tracer le contour du cytoplasme en 20 points");
        run("Add to Manager");
        run("Measure");
        Area_Cyto=getResult("Area");
        RawIntDen_Cyto=getResult("RawIntDen");
        IntDen_Cyto=getResult("IntDen");

        t0 = roiManager("count");
        roiManager("Select", t0-1);
        ZoneCyto = roiManager("index");
        getSelectionCoordinates(X_cytoplasme, Y_cytoplasme);

        makePolygon(X_cytoplasme[0], Y_cytoplasme[0], X_cytoplasme[1],
Y_cytoplasme[1],X_cytoplasme[2], Y_cytoplasme[2], Xm1/ww, Ym1/ww, X_cytoplasme[18],
Y_cytoplasme[18], X_cytoplasme[19], Y_cytoplasme[19]);
        run("Add to Manager");
        roiManager("Select", t0);
        roiManager("Rename", "Zone 1");
        Zone1 = roiManager("index");

        makePolygon(X_cytoplasme[18], Y_cytoplasme[18], Xm1/ww, Ym1/ww, X_cytoplasme[14],
Y_cytoplasme[14], X_cytoplasme[15], Y_cytoplasme[15], X_cytoplasme[16],
Y_cytoplasme[16],X_cytoplasme[17], Y_cytoplasme[17]);
        run("Add to Manager");
        roiManager("Select", t0+1);
        roiManager("Rename", "Zone 2");
        Zone2 = roiManager("index");

        makePolygon(X_cytoplasme[2], Y_cytoplasme[2], X_cytoplasme[3], Y_cytoplasme[3],
X_cytoplasme[4], Y_cytoplasme[4], Xm2/ww, Ym2/ww, Xm1/ww, Ym1/ww);
        run("Add to Manager");
        roiManager("Select", t0+2);
        roiManager("Rename", "Zone 3");
        Zone3 = roiManager("index");

        makePolygon(Xm1/ww, Ym1/ww, Xm2/ww, Ym2/ww, X_cytoplasme[12], Y_cytoplasme[12],
X_cytoplasme[13], Y_cytoplasme[13], X_cytoplasme[14], Y_cytoplasme[14]);
        run("Add to Manager");
        roiManager("Select", t0+3);
        roiManager("Rename", "Zone 4");
        Zone4 = roiManager("index");

        makePolygon(X_cytoplasme[4], Y_cytoplasme[4], X_cytoplasme[5], Y_cytoplasme[5],
X_cytoplasme[6], Y_cytoplasme[6], X_cytoplasme[7], Y_cytoplasme[7], X_cytoplasme[8],
Y_cytoplasme[8], Xm2/ww, Ym2/ww);
        run("Add to Manager");
        roiManager("Select", t0+4);
        roiManager("Rename", "Zone 5");
        Zone5 = roiManager("index");

        makePolygon(Xm2/ww, Ym2/ww, X_cytoplasme[8], Y_cytoplasme[8], X_cytoplasme[9],
Y_cytoplasme[9], X_cytoplasme[10], Y_cytoplasme[10], X_cytoplasme[11], Y_cytoplasme[11],
X_cytoplasme[12], Y_cytoplasme[12]);
        run("Add to Manager");
        roiManager("Select", t0+5);
        roiManager("Rename", "Zone 6");
        Zone6 = roiManager("index");

        roiManager("Show All without labels");

//********************************************************************ANALYSE DE LA MARGE
ANTERIEUR ********************************************************************************
```

```
if (cortex == true){
	setTool("polygon");
	waitForUser("Choisis la zone Marge Anterieur");
	run("Add to Manager");
	ZoneMA = roiManager("index");
	t0 = roiManager("count");
	run("Clear Results");
	run("Duplicate...","title=Marge-Anterieur duplicate channels=1");
	MA = getImageID();
	setBackgroundColor(0, 0, 0);
	run("Clear Outside");
	selectWindow("Marge-Anterieur");
	run("Duplicate...","title=Marge-Anterieur-1 duplicate channels=1");
	MA1 = getImageID();
	//valeur 10 de threshold avant
	setThreshold(3, 255);
	run("Convert to Mask");
	run("Analyze Particles...", "size="+ww*ww*2+"-Infinity circularity=0.00-1.00 
show=[Overlay Outlines] display include summarize add in_situ");
	roiManager("Show All")
	nb_Zone_MA=nResults;
	t1 = roiManager("count");
	run("Clear Results");
	Area_MA=0;
	RawIntDen_MA=0;
	IntDen_MA=0;
	for (i=t0; i<=t1-1;i++){
		selectImage(MA);
		roiManager("Select", i);
		run("Measure");
		Area_MA = Area_MA+getResult("Area");
		RawIntDen_MA = RawIntDen_MA+getResult("RawIntDen");
		IntDen_MA = IntDen_MA+getResult("IntDen");
		roiManager("Rename", "ZoneMA"+i-t0+1);
		roiManager("UseNames", "true");
		roiManager("Show All with labels");
	}
	run("Summarize");

	// permet de savoir selon la valeur de mean si il y a une ou plusieurs zones 
d'identifiées, et pourquoi pas mettre juste la valeur du compteur de zones ?
	if (nResults > 1){
		Mean_MA = getResult("Mean",nb_Zone_MA);
		SD_Mean_MA = getResult("Mean",nb_Zone_MA+1);
	}
	else if (nResults == 1){
		Mean_MA = getResult("Mean",0);
		SD_Mean_MA = 0;
	}
	else if (nResults == 0){
		Mean_MA = 0;
		SD_Mean_MA = 0;
		Area_MA = 0;
		RawIntDen_MA = 0;
		IntDen_MA = 0;
	}

//****************************************************************ANALYSE DE LA MEMBRANE AP
********************************************************************************

	setTool("polygon");
	waitForUser("Choisis la zone Membrane Lateral A");
	run("Add to Manager");
	ZoneAP = roiManager("index");
	t0 = roiManager("count");
	run("Clear Results");
	run("Duplicate...","title=Membrane-AP duplicate channels=1");
	Membrane_AP = getImageID();
	setBackgroundColor(0, 0, 0);
	run("Clear Outside");
	selectWindow("Membrane-AP");
	run("Duplicate...","title=Membrane-AP-1 duplicate channels=1");
	Membrane_AP1 = getImageID();
	//valeur 10 de threshold avant
	setThreshold(3, 255);
	run("Convert to Mask");
	run("Analyze Particles...", "size="+ww*ww*2+"-Infinity circularity=0.00-1.00 
show=[Overlay Outlines] display include summarize add in_situ");
```

```
        roiManager("Show All with labels");
        roiManager("Show All");
        nb_Zone_Membrane_AP = nResults;
        t1 = roiManager("count");
        run("Clear Results");
        Area_Membrane_AP=0;
        RawIntDen_Membrane_AP=0;
        IntDen_AP=0;
        for (i=t0; i<=t1-1;i++){
               selectImage(Membrane_AP);
               roiManager("Select", i);
               run("Measure");
               Area_Membrane_AP=Area_Membrane_AP+getResult("Area");
               RawIntDen_Membrane_AP=RawIntDen_Membrane_AP+getResult("RawIntDen");
               IntDen_Membrane_AP=IntDen_Membrane_AP+getResult("IntDen");
               roiManager("Rename", "ZoneMembraneAP"+i-t0+1);
               roiManager("UseNames", "true");
               roiManager("Show All with labels");
               }
        run("Summarize");
        if (nResults > 1){
               Mean_Membrane_AP = getResult("Mean",nb_Zone_Membrane_AP);
               SD_Mean_Membrane_AP = getResult("Mean",nb_Zone_Membrane_AP+1);
        }
        else if (nResults == 1){
               Mean_Membrane_AP = getResult("Mean",0);
               SD_Mean_Membrane_AP = 0;
        }
        else if (nResults == 0){
               Mean_Membrane_AP = 0;
               SD_Mean_Membrane_AP = 0;
               Area_Membrane_AP = 0;
               RawIntDen_Membrane_AP = 0;
        }

//*******************************************************************ANALYSE DE LA MEMBRANE BP
********************************************************************************

        setTool("polygon");
        waitForUser("Choisis la zone Membrane Lateral B");
        run("Add to Manager");
        ZoneBP = roiManager("index");
        t0 = roiManager("count");
        run("Clear Results");
        run("Duplicate...","title=Membrane-BP duplicate channels=1");
        Membrane_BP = getImageID();
        setBackgroundColor(0, 0, 0);
        run("Clear Outside");
        selectWindow("Membrane-BP");
        run("Duplicate...","title=Membrane-BP-1 duplicate channels=1");
        Membrane_BP1 = getImageID();
        //valeur 10 de threshold avant
        setThreshold(3, 255);
        run("Convert to Mask");
        run("Analyze Particles...", "size="+ww*ww*2+"-Infinity circularity=0.00-1.00
show=[Overlay Outlines] display include summarize add in_situ");
        roiManager("Show All with labels");
        roiManager("Show All");
        nb_Zone_Membrane_BP=nResults;
        t1 = roiManager("count");
        run("Clear Results");
        Area_Membrane_BP=0;
        RawIntDen_Membrane_BP=0;
        IntDen_BP=0;
        for (i=t0; i<=t1-1;i++){
               selectImage(Membrane_BP);
               roiManager("Select", i);
               run("Measure");
               Area_Membrane_BP=Area_Membrane_BP+getResult("Area");
               RawIntDen_Membrane_BP=RawIntDen_Membrane_BP+getResult("RawIntDen");
               IntDen_Membrane_BP=IntDen_Membrane_BP+getResult("IntDen");
               roiManager("Rename", "ZoneMembraneBP"+i-t0+1);
               roiManager("UseNames", "true");
               roiManager("Show All with labels");
        }
        run("Summarize");
        if (nResults > 1){
               Mean_Membrane_BP = getResult("Mean",nb_Zone_Membrane_BP);
               SD_Mean_Membrane_BP = getResult("Mean",nb_Zone_Membrane_BP+1);
```

```
	}
	else if (nResults == 1){
		Mean_Membrane_BP = getResult("Mean",0);
		SD_Mean_Membrane_BP = 0;
	}
	else if (nResults == 0){
		Mean_Membrane_BP = 0;
		SD_Mean_Membrane_BP = 0;
		Area_Membrane_BP=0;
		RawIntDen_Membrane_BP=0;
	}

//***********************ANALYSE DE LA MEMBRANE POSTERIEUR ****************************

	setTool("polygon");
	waitForUser("Choisis la zone Posterieur");
	run("Add to Manager");
	ZoneP = roiManager("index");
	t0 = roiManager("count");
	run("Clear Results");
	run("Duplicate...","title=Posterieur duplicate channels=1");
	Posterieur = getImageID();
	setBackgroundColor(0, 0, 0);
	run("Clear Outside");
	selectWindow("Posterieur");
	run("Duplicate...","title=Posterieur-1 duplicate channels=1");
	Posterieur1 = getImageID();
	//valeur 10 de threshold avant
	setThreshold(3, 255);
	run("Convert to Mask");
	run("Analyze Particles...", "size="+ww*ww*2+"-Infinity circularity=0.00-1.00
show=[Overlay Outlines] display include summarize add in_situ");
	roiManager("Show All");
	nb_Zone_posterieur=nResults;
	t1 = roiManager("count");
	run("Clear Results");
	Area_posterieur=0;
	RawIntDen_posterieur=0;
	IntDen_posterieur=0;
	for (i=t0; i<=t1-1;i++){
		selectImage(Posterieur);
		roiManager("Select", i);
		run("Measure");
		Area_posterieur=Area_posterieur+getResult("Area");
		RawIntDen_posterieur=RawIntDen_posterieur+getResult("RawIntDen");
		IntDen_posterieur=IntDen_posterieur+getResult("IntDen");
		roiManager("Rename", "ZonePosterieur"+i-t0+1);
		roiManager("UseNames", "true");
		roiManager("Show All with labels");
	}
	run("Summarize");
	if (nResults > 1){
		Mean_posterieur = getResult("Mean",nb_Zone_posterieur);
		SD_Mean_posterieur = getResult("Mean",nb_Zone_posterieur+1);
	}
	else if (nResults == 1){
		Mean_posterieur = getResult("Mean",0);
		SD_Mean_posterieur = 0;
	}
	else if (nResults == 0){
		Mean_posterieur = 0;
		SD_Mean_posterieur = 0;
		Area_posterieur=0;
		RawIntDen_posterieur=0;
	}

	run("Clear Results");
	setResult("Area",0,Area_MA);
	setResult("Nombres de zones",0,nb_Zone_MA);
	//setResult("Mean intensity non significatif",0,Mean_MA);
	//setResult("SD Mean",0,SD_Mean_MA);
	setResult("Somme nvx de gris rapportée aux pixels",0,RawIntDen_MA);
	setResult("Somme nvx de gris rapportée aux µm^2",0,IntDen_MA);
	setResult("Densite (nvx de gris/µm^2)",0,IntDen_MA/Area_MA);

	setResult("Area",1,Area_Membrane_AP);
	setResult("Nombres de zones",1,nb_Zone_Membrane_AP);
	setResult("Somme nvx de gris rapportée aux pixels",1,RawIntDen_Membrane_AP);
	setResult("Somme nvx de gris rapportée aux µm^2",1,IntDen_Membrane_AP);
```

```
        setResult("Densite (nvx de gris/µm^2)",1,IntDen_Membrane_AP/Area_Membrane_AP);

        setResult("Area",2,Area_Membrane_BP);
        setResult("Nombres de zones",2,nb_Zone_Membrane_BP);
        setResult("Somme nvx de gris rapportée aux pixels",2,RawIntDen_Membrane_BP);
        setResult("Somme nvx de gris rapportée aux µm^2",2,IntDen_Membrane_BP);
        setResult("Densite (nvx de gris/µm^2)",2,IntDen_Membrane_BP/Area_Membrane_BP);

        setResult("Area",3,Area_posterieur);
        setResult("Nombres de zones",3,nb_Zone_posterieur);
        setResult("Somme nvx de gris rapportée aux pixels",3,RawIntDen_posterieur);
        setResult("Somme nvx de gris rapportée aux µm^2",3,IntDen_posterieur);
        setResult("Densite (nvx de gris/µm^2)",3,IntDen_posterieur/Area_posterieur);

        setResult("Area",4,Area_Cyto);
        setResult("Nombres de zones",4,1);
        setResult("Somme nvx de gris rapportée aux pixels",4,RawIntDen_Cyto);
        setResult("Somme nvx de gris version SC",4,IntDen_Cyto);
        setResult("Densite (nvx de gris/µm^2)",4,IntDen_Cyto/Area_Cyto);

        updateResults;

        saveAs("measurements", ""+dir+"Ovocyte "+num_ovo+" Zones.xls");
}

//*****************************************************************************ETUDE DU
PROFIL DES MEMBRANES**********************************************************************

if (profil == true){
// Profil membrane AP:
        setTool("polyline");
        run("Line Width...", "line="+4/ww+"");
        waitForUser("Tracer la ligne de A à P!!!");
        run("Add to Manager");
        run("Clear Results");
        profile_AP = getProfile();
        for (j=0; j<profile_AP.length; j++){
                setResult("Membrane AP", j, profile_AP[j]);
        }

// Profil membrane PB:
        setTool("polyline");
        run("Line Width...", "line="+4/ww+"");
        waitForUser("Tracer la ligne de P à B et non l'inverse !!!");
        run("Add to Manager");
        profile_PB = getProfile();
        for (j=0; j<profile_PB.length; j++){
                setResult("Membrane PB", j, profile_PB[j]);
        }

// Profil membrane MA:
        setTool("polyline");
        run("Line Width...", "line="+4/ww+"");
        waitForUser("Tracer la ligne marge anterieur");
        run("Add to Manager");
        profile_MA = getProfile();
        for (j=0; j<profile_MA.length; j++){
                setResult("Membrane MA", j, profile_MA[j]);
        }

  // Profil membrane Post:
        setTool("polyline");
        run("Line Width...", "line="+4/ww+"");
        waitForUser("Tracer la ligne posterieur");
        run("Add to Manager");
        profile_Post = getProfile();


// Profil de deux membranes de cellule nourricière:

        setTool("polyline");
        run("Line Width...", "line="+4/ww+"");
        waitForUser("Tracer une membrane simple d'une cellule nourricière");
        run("Add to Manager");
        profile_NC1 = getProfile();
        for (j=0; j<profile_NC1.length; j++){
                setResult("Membrane Cellule Nourriciere simple", j, profile_NC1[j]);
        }
        waitForUser("Tracer une  membrane double d'une cellule nourriciere");
```

```
        run("Add to Manager");
        profile_NC2 = getProfile();
        for (j=0; j<profile_NC2.length; j++){
                setResult("Membrane Cellule Nourriciere double", j, profile_NC2[j]);
        }
//Affiche les longueurs de membrane anterieur et nurse cells
        setResult("Longueur MA",0,profile_MA.length);
        setResult("Longueur NC1",0,profile_NC1.length);
        setResult("Longueur NC2",0,profile_NC2.length);
        setResult("Longueur AP",0,profile_AP.length);
        setResult("Longueur PB",0,profile_PB.length);
        setResult("Longueur Post",0,profile_Post.length);
        setResult("Taille pixel",0,ww);

// Affiche les profiles dans la fenêtre résultat et les sauve:
        updateResults;
        saveAs("measurements", ""+dir+"Ovocyte "+num_ovo+" Profil intensite membranes.xls");
        run("Clear Results");
}

//*****************************************************************************************ETUDE
DU CYTOPLASME*****************************************************************************

if (cytoplasme == true){
        selectImage("Image3");
        run("Duplicate...","title=Image_de_depart_dupliquee duplicate channels=1");
        roiManager("select", ZoneCyto);
        run("Clear Outside");


        //Zone 1 :
        run("Clear Results");
        IntDen_Total_Z1 = 0;
        selectWindow("Image_de_depart_dupliquee");
        roiManager("select",Zone1);
        run("Measure");
        Area_Z1 = getResult("Area");
        IntDen_Total_Z1 = getResult("IntDen");
        run("Duplicate...","title=Zone1 duplicate channels=1");
        run("Clear Outside");
        Image_Zone1 = getImageID();

// 3D object counter
        run("Clear Results");
        run("3D OC Options", "  surface nb_of_obj._voxels mean_gray_value dots_size=5
font_size=10 show_numbers white_numbers
store_results_within_a_table_named_after_the_image_(macro_friendly) redirect_to=none");
        run("3D Objects Counter", "threshold=1 slice=0 min.=10 max.=1048576 objects
statistics");
        selectWindow("Statistics for Zone1");
        saveAs("Results", ""+dir+"Ovocyte "+num_ovo+" particules Z1.xls");

        //Zone 2 :
        run("Clear Results");
        IntDen_Total_Z2 = 0;
        selectWindow("Image_de_depart_dupliquee");
        roiManager("select",Zone2);
        run("Measure");
        Area_Z2 = getResult("Area");
        IntDen_Total_Z2 = getResult("IntDen");
        run("Duplicate...","title=Zone2 duplicate channels=1");
        run("Clear Outside");
        Image_Zone2 = getImageID();

        // 3D object counter
        run("Clear Results");
        run("3D OC Options", "  surface nb_of_obj._voxels mean_gray_value dots_size=5
font_size=10 show_numbers white_numbers
store_results_within_a_table_named_after_the_image_(macro_friendly) redirect_to=none");
        run("3D Objects Counter", "threshold=1 slice=0 min.=10 max.=1048576 objects
statistics");
        selectWindow("Statistics for Zone2");
        saveAs("Results", ""+dir+"Ovocyte "+num_ovo+" particules Z2.xls");


        //Zone 3 :
        run("Clear Results");
        IntDen_Total_Z3 = 0;
        selectWindow("Image_de_depart_dupliquee");
```

```
        roiManager("select",Zone3);
        run("Measure");
        Area_Z3 = getResult("Area");
        IntDen_Total_Z3 = getResult("IntDen");
        run("Duplicate...","title=Zone3 duplicate channels=1");
        run("Clear Outside");
        Image_Zone3 = getImageID();

// 3D object counter
        run("Clear Results");
        run("3D OC Options", "  surface nb_of_obj._voxels mean_gray_value dots_size=5
font_size=10 show_numbers white_numbers
store_results_within_a_table_named_after_the_image_(macro_friendly) redirect_to=none");
        run("3D Objects Counter", "threshold=1 slice=0 min.=10 max.=1048576 objects
statistics");
        selectWindow("Statistics for Zone3");
        saveAs("Results", ""+dir+"Ovocyte "+num_ovo+" particules Z3.xls");


        //Zone 4 :
        run("Clear Results");
        IntDen_Total_Z4 = 0;
        selectWindow("Image_de_depart_dupliquee");
        roiManager("select",Zone4);
        run("Measure");
        Area_Z4 = getResult("Area");
        IntDen_Total_Z4 = getResult("IntDen");
        run("Duplicate...","title=Zone4 duplicate channels=1");
        run("Clear Outside");
        Image_Zone4 = getImageID();

        // 3D object counter
        run("Clear Results");
        run("3D OC Options", "  surface nb_of_obj._voxels mean_gray_value dots_size=5
font_size=10 show_numbers white_numbers
store_results_within_a_table_named_after_the_image_(macro_friendly) redirect_to=none");
        run("3D Objects Counter", "threshold=1 slice=0 min.=10 max.=1048576 objects
statistics");
        selectWindow("Statistics for Zone4");
        saveAs("Results", ""+dir+"Ovocyte "+num_ovo+" particules Z4.xls");


        //Zone 5:
        run("Clear Results");
        IntDen_Total_Z5 = 0;
        selectWindow("Image_de_depart_dupliquee");
        roiManager("select",Zone5);
        run("Measure");
        Area_Z5 = getResult("Area");
        IntDen_Total_Z5 = getResult("IntDen");
        run("Duplicate...","title=Zone5 duplicate channels=1");
        run("Clear Outside");
        Image_Zone5 = getImageID();

        // 3D object counter
        run("Clear Results");
        run("3D OC Options", "  surface nb_of_obj._voxels mean_gray_value dots_size=5
font_size=10 show_numbers white_numbers
store_results_within_a_table_named_after_the_image_(macro_friendly) redirect_to=none");
        run("3D Objects Counter", "threshold=1 slice=0 min.=10 max.=1048576 objects
statistics");
        selectWindow("Statistics for Zone5");
        saveAs("Results", ""+dir+"Ovocyte "+num_ovo+" particules Z5.xls");


        //Zone 6 :
        run("Clear Results");
        IntDen_Total_Z6 = 0;
        selectWindow("Image_de_depart_dupliquee");
        roiManager("select",Zone6);
        run("Measure");
        Area_Z6 = getResult("Area");
        IntDen_Total_Z6 = getResult("IntDen");
        run("Duplicate...","title=Zone6 duplicate channels=1");
        run("Clear Outside");
        Image_Zone6 = getImageID();

        // 3D object counter
        run("Clear Results");
```

```
        run("3D OC Options", "  surface nb_of_obj._voxels mean_gray_value dots_size=5
font_size=10 show_numbers white_numbers
store_results_within_a_table_named_after_the_image_(macro_friendly) redirect_to=none");
        run("3D Objects Counter", "threshold=1 slice=0 min.=10 max.=1048576 objects
statistics");
        selectWindow("Statistics for Zone6");
        saveAs("Results", ""+dir+"Ovocyte "+num_ovo+" particules Z6.xls");


showMessage("C'est fini !");
if (z!=true){
        //close("*");
        selectWindow("Image3");
        close("\\Others");
        selectWindow("Results");
        run("Close");
        selectWindow("Summary");
        run("Close");
}
}
```
